# Association of the TREM‐1–STAT3 Axis With Stroke Severity and Functional Outcomes in Ischemic Stroke: A Prospective Case‐Control Study

**DOI:** 10.1002/brb3.71633

**Published:** 2026-08-05

**Authors:** Haohai Lin, Yan Dong, Zhangping He, Chao Qin, Pingping Li

**Affiliations:** ^1^ Department of Neurology The First Affiliated Hospital of Guangxi Medical University Nanning China

**Keywords:** acute ischemic stroke, prognosis, STAT3, TREM‐1

## Abstract

**Background:**

Information regarding the signaling axis involving triggering receptor expressed on myeloid cells 1 (TREM‐1) and its downstream mediator, signal transducer and activator of transcription 3 (STAT3), remains limited in ischemic stroke. We investigated the plasma levels of TREM‐1 in patients with ischemic stroke and compared to controls. Second, we investigated the potential correlation between plasma TREM‐1 levels and STAT3 expression in peripheral blood mononuclear cells (PBMCs) and explored their associations with stroke severity and functional outcomes.

**Methods:**

In an observational case‐control study, plasma and PBMCs from participants were collected to measure TREM‐1 and STAT3 expression levels. The National Institutes of Health Stroke Scale (NIHSS) at admission, day 7, and 1 and 3 months, and the modified Rankin Scale (mRS) at 3 months post‐discharge were assessed. Plasma TREM‐1 levels and STAT3 expression were measured by enzyme‐linked immunosorbent assay (ELISA) and quantitative reverse transcription polymerase chain reaction (RT‐qPCR), respectively. The Inflammation‐Related Principal Component Score (IRPCS) was examined for its association with 3‐month functional outcome.

**Result:**

TREM‐1 and STAT3 were significantly elevated in acute ischemic stroke (AIS) patients (*p* < 0.05, *p* < 0.0001) and positively correlated (ρ = 0.432, *p* < 0.0001). Both biomarkers correlated with the NIHSS at 1 and 3 months and mRS at 3 months (all *p* < 0.05). The composite IRPCS showed the strongest association with poor 3‐month functional outcome among the tested biomarkers (OR = 2.84, 95% CI: 1.32–6.12, *p* = 0.008).

**Conclusion:**

Plasma TREM‐1 levels were positively correlated with STAT3 expression. The TREM‐1–STAT3 axis was associated with stroke severity and 3‐month functional outcome in AIS patients. These findings should be regarded as exploratory and require validation in larger cohorts.

## Graphical abstract

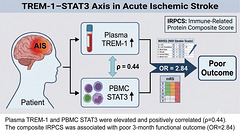

**Abbreviations**
AIS: acute ischemic stroke
cDNA: complementary DNA
EDTA: ethylenediaminetetraacetic acid
ELISA: enzyme‐linked immunosorbent assay
INR: international normalized ratio
IRPCS: inflammation‐related principal component score
mRS: modified Rankin Scale
NIHSS: National Institutes of Health Stroke Scale
OR: odds ratio
PBMCs: peripheral blood mononuclear cells
PCA: principal component analysis
ROC: receiver operating characteristic
RT‐qPCR: quantitative reverse transcription polymerase chain reaction
STAT3: signal transducer and activator of transcription 3
TREM‐1: triggering receptor expressed on myeloid Cells 1

## 1 Introduction

By 2021, stroke ranked as the fourth prominent driver of disability alongside the third primary source of fatality worldwide (Feigin et al. 2024). Across the world, the census of individuals surviving with post‐stroke impairments or dying from this condition has escalated drastically between 1990 and 2021 (Tu et al. 2023). Pathophysiologically, an interruption in blood perfusion yields a cerebral microenvironment characterized by hypoxia. This severe deprivation directly compromises brain microvascular endothelial cells (BMECs), ultimately culminating in the disruption of the blood–brain barrier (BBB) (Z. Yang et al. 2021). In the setting of acute cerebral ischemia, the compromised and necrotic tissue undergoes cell death, triggering the massive liberation of damage‐associated molecular patterns (DAMPs). Circulating monocytes give rise to Monocyte‑derived macrophages (MDMs) that detect these stress signals and DAMPs, promptly turning activated before infiltrating into the focal site of tissue damage (Castellanos‐Molina et al. 2024; M. Yang et al. 2025; C. Li et al. 2021). This initiates innate and adaptive immune responses that unfold over days to weeks after the stroke (Alsbrook et al. 2023).

As a key transmembrane immune receptor prominently localized on the surface of phagocytes, triggering receptor expressed on myeloid cells 1 (TREM‐1) functions as a potent amplifier of secondary inflammatory cascades. Heightened levels of circulating TREM‐1 have been documented in individuals suffering from acute myocardial infarction, directly tracking with a poorer clinical prognosis (C. Li et al. 2024; Trivedi et al. 2025; Theobald et al. 2024; Ait‐Oufella et al. 2021). Parallel to this, elevated TREM‐1 concentrations within cerebrospinal fluid have been linked to exacerbated disease severity and delayed ischemic complications among patients diagnosed with subarachnoid hemorrhage (Kapapa et al. 2025). Following cerebral infarction, elevated plasma TREM‐1 levels have been shown to correlate with patient prognosis (Backes et al. 2021), indicating a critical role for TREM‐1 in the inflammatory cascade after ischemic stroke. Signal transducer and activator of transcription (STAT) proteins are a family of cytoplasmic transcription factors with conserved modular structures. Among them, STAT3 is a central mediator of cytokine‐driven immune responses (Darnell et al. 1994; Lodi et al. 2022; Hajialigol et al. 2025). In patients with myocardial infarction, both elevated plasma TREM‐1 levels and increased STAT3 expression in peripheral blood mononuclear cells (PBMCs) are associated with adverse clinical outcomes (Boufenzer et al. 2015; Zhao et al. 2011). Mechanistically, TREM‐1 is an innate immune receptor mainly expressed on myeloid‐lineage cells. Upon activation, TREM‐1 signals through the adaptor protein DAP12/TYROBP and amplifies inflammatory kinase cascades, including Syk‐ and MAPK‐related pathways (QIAGEN n.d.). Experimental studies have suggested that TREM‐1‐related signaling may converge on ERK1/2–STAT3 or STAT3/HIF‐1α pathways in inflammatory and neuroinflammatory models (Bhusal et al. 2024; Wu et al. 2022). These findings provide biological plausibility for examining TREM‐1 and STAT3 together as an inflammation‐related axis in ischemic stroke.

## 2 Materials and Methods

### 2.1 Study Design

This study utilized a prospective cohort design. A cross‐sectional analysis was conducted at baseline—upon hospital admission—to compare TREM‐1 and STAT3 levels between patients with acute ischemic stroke (AIS) and control subjects. Thereafter, a 3‐month longitudinal follow‐up was carried out in the AIS cohort to examine the association between baseline biomarker levels and long‐term neurological outcomes.

### 2.2 Participants

For this investigation, participating patients were restricted to those with ischemic stroke. Furthermore, their diagnoses had to be established by magnetic resonance imaging (MRI) within 7 days of the initial symptom onset. All participants were admitted to the Neurology Department of The First Affiliated Hospital of Guangxi Medical University between July 2024 and February 2025. Subjects meeting any of the following conditions were disqualified from the study: (1) pediatric patients (<18 years old) or those unable to undergo MRI; (2) a history of dementia, cranial trauma, primary intracerebral hemorrhage, or a recent stroke within 90 days; (3) systemic or severe pathologies, such as active tumors, sepsis, renal insufficiency, heart failure, or infections of the central nervous system; and (4) ongoing active inflammatory or autoimmune conditions. Furthermore, women who were lactating or pregnant, alongside individuals with potential follow‐up barriers, were excluded. The control subjects were recruited from patients with stroke‐unrelated neurological disorders admitted to the same neurology wards during the same enrollment period (July 2024–February 2025). Control diagnoses included benign paroxysmal positional vertigo (BPPV, *n* = 9), persistent postural‐perceptual dizziness (PPPD, *n* = 37), tension‐type headache (TTH, *n* = 4), and Ménière's Disease (MD, *n* = 9). Exclusion criteria for controls were identical to those for the stroke group, including active inflammatory or autoimmune disease, acute infection, malignancy, and recent stroke.

### 2.3 Clinical Data Collection and Follow‐up

Upon admission, all patients received standard clinical care. The general characteristics of all patients were collected as part of the clinical history (e.g., age, sex, risk factors, past medical history). Laboratory parameters, including white blood cell count, platelet count, hemoglobin, lipid profile (total cholesterol and triglycerides), international normalized ratio (INR), D‐dimer, and high‐sensitivity C‐reactive protein (hs‐CRP), were collected from all patients. In the stroke group, MRI images were obtained to support the diagnosis of AIS, and neurological severity was assessed using the National Institutes of Health Stroke Scale (NIHSS) score at admission, day 7, 1 month, and 3 months post‐discharge to evaluate the neurological severity. Functional prognosis was also evaluated with the modified Rankin Scale (mRS) at 3 months post‐discharge. All follow‐up assessments were conducted by trained personnel who were blinded to the laboratory biomarker results.

### 2.4 Plasma Sample Collected for Enzyme‐Linked Immunosorbent Assay

Peripheral venous blood (5 mL) was drawn into ethylenediaminetetraacetic acid (EDTA)‐anticoagulated tubes within 24 h of admission. Platelet‐free plasma was obtained by centrifugation (1200 × *g*, 15 min, 4°C), immediately frozen, and stored at −80°C. Plasma TREM‐1 concentrations were quantified in triplicate using a commercial enzyme‐linked immunosorbent assay (ELISA) kit (E‐EL‐H6177; Elabscience, Wuhan, China), following the manufacturer's protocol with instrument calibration performed against the factory standard curve.

### 2.5 PBMCs Isolation and Quantitative Reverse Transcription Polymerase Chain Reaction

For the isolation process, phosphate‐buffered saline (PBS) was utilized to dilute fresh peripheral blood at a 1:1 ratio. In sterile centrifuge tubes, this diluted specimen was carefully placed over Ficoll‐Paque PREMIUM (1.077 g/mL; cat. no. 17‐5442‐02) in a 1:1 volumetric proportion. Centrifugation was carried out for 20 min at 400 × *g* (room temperature), after which the mononuclear cell layer was precisely aspirated and moved to a clean tube. Total RNA was extracted from harvested cell samples using an RNAeasy kit (R0026; Beyotime Biotechnology). RNA was reverse‐transcribed to cDNA with a first‐strand cDNA synthesis kit (B532445; Sangon Biotech, Shanghai, China), per the manufacturer's instructions. Quantitative reverse transcription polymerase chain reaction (RT‐qPCR) was performed on an Applied Biosystems 7500 Fast Real‐Time PCR System with a 96‐well block, using SYBR‐Green assays. Relative expression data were analyzed via the 2‐ΔΔCt approach and normalized to β‐actin. STAT3 amplification primers were as follows: 5′‐GGGCTTCTCCTTCTGGGTCTG‐3′(fwd)and 5′‐CCGCTCCCGCTCCTTACTG‐3′ (rev).

### 2.6 Statistical Analysis

All statistical evaluations were conducted using SPSS Statistics version 26.0 (IBM Corp., Armonk, NY, USA). A two‐tailed *p* value <0.05 was defined as the threshold for statistical significance. Categorical variables are presented as counts and percentages. The normality of continuous variable distributions was verified using the Shapiro–Wilk test. Normally distributed baseline data are reported as the mean ± standard deviation (SD), while other experimental or clinical outcomes are expressed as the mean ± standard error of the mean (SEM). Non‐normally distributed data are expressed as median and interquartile range (IQR). Comparisons between the groups were performed using the Student's *t*‐test for normally distributed variables, the Mann–Whitney *U* test for non‐normally distributed variables, and the chi‐squared (*χ* ^2^) test for categorical variables. Given that NIHSS and mRS scores are ordinal variables, Spearman's rank correlation coefficient was used for all bivariate correlation analyses. Binary logistic regression was employed to evaluate the predictive ability of individual biomarkers and their composite score for neurological outcomes, with model performance assessed by the area under the receiver operating characteristic curve (AUC), sensitivity, specificity, and Nagelkerke *R* ^2^. Principal component analysis (PCA) was conducted after confirming data suitability via the Kaiser–Meyer–Olkin measure and Bartlett's test of sphericity, and the resulting component scores were used as a composite variable in subsequent logistic regression analyses.

## 3 Results

### 3.1 Baseline Characteristics

A total of 96 patients with acute ischemic stroke and 57 control subjects were enrolled. The demographic characteristics, risk factors, and laboratory values of the enrolled subjects are presented in Table 1. Demographically, the median age was comparable between the control group (64 years; IQR 53–68) and the stroke group (61 years; IQR 57–66; *p* = 0.956). Although a higher proportion of males was observed in the stroke group (68.8%) compared to the control group (54.4%), this difference did not reach statistical significance (*p* = 0.075). Regarding clinical risk factors, the prevalence of hypertension (70.0% vs. 64.9%, *p* = 0.532), diabetes mellitus (30.2% vs. 28.1%, *p* = 0.779), smoking history (36.5% vs. 26.3%, *p* = 0.216), and alcohol use history (44.0% vs. 31.6%, *p* = 0.136) did not differ significantly between the stroke and control groups. Key hematological and biochemical markers, including white blood cell count (7.56 ± 1.81 vs. 7.24 ± 2.52 ×10^9^/L, *p* = 0.409), platelet count (*p* = 0.946), hemoglobin (138.6 ± 15.58 vs. 133.6 ± 16.38 g/L, *p* = 0.055), and lipid profiles (total cholesterol: 4.90 ± 1.05 vs. 4.60 ± 1.36 mmol/L, *p* = 0.175; triglycerides: *p* = 0.120), all showed no statistically significant differences. Similarly, coagulation and inflammation markers, namely INR (*p* = 0.128), D‐dimer (*p* = 0.485), and hs‐CRP (*p* = 0.964), were not significantly different between the two cohorts. Thus, statistical analysis revealed no significant differences between the two groups at baseline, indicating that the subjects in each group were comparable.

**TABLE 1:** Comparison of baseline characteristics.

Characteristic	Control group (*n* = 57)	Stroke group (*n* = 96)	Statistic	*p* value
Demographics				
Age (years)	64 (53, 68)	61 (57, 66)	*U* = 2721.5	0.956
Sex (male)	54.40%	68.80%	*χ* ^2^ = 3.18	0.075
Risk factors				
Smoking history	26.30%	36.50%	*χ* ^2^ = 1.672	0.216
Alcohol history	31.60%	44.00%	*χ* ^2^ = 2.223	0.136
Diabetes mellitus	28.10%	30.20%	*χ* ^2^ = 0.079	0.779
Hypertension	64.90%	70.00%	*χ* ^2^ = 0.391	0.532
Laboratory values				
White blood cell (×10^9^/L)	7.24 ± 2.52	7.56 ± 1.81	*t* = −0.829, df = 151	0.409
Platelet count (×10^9^/L)	230.4 (201.7, 260.8)	232.0 (191.5, 270.5)	*U* = 2718	0.946
Hemoglobin (g/L)	133.6 ± 16.38	138.6 ± 15.58	*t* = −1.913, df = 151	0.055
Total cholesterol (mmol/L)	4.60 ± 1.36	4.90 ± 1.05	*t* = −1.368, df = 151	0.175
Triglycerides (mmol/L)	1.41 (1.10, 2.22)	1.40 (1.12, 1.67)	*U* = 2147.5	0.12
INR	1.04 (0.99, 1.09)	1.02 (0.96, 1.08)	*U* = 2307.5	0.128
D‐Dimer (mg/L)	108 (67, 229)	122.5 (87, 183)	*U* = 2180.5	0.485
hs‐CRP (mg/L)	1.15 (0, 3.31)	0.6 (0, 4.1)	*U* = 1234.5	0.964

*Note*: Data are presented as mean ± standard deviation, median [interquartile range], or %.

Abbreviations: hs‐CRP, high‐sensitivity C‐reactive protein; INR, international normalized ratio.

### 3.2 Elevated TREM‐1 Levels and STAT3 Expression in Stroke Patients

**FIGURE 1:**
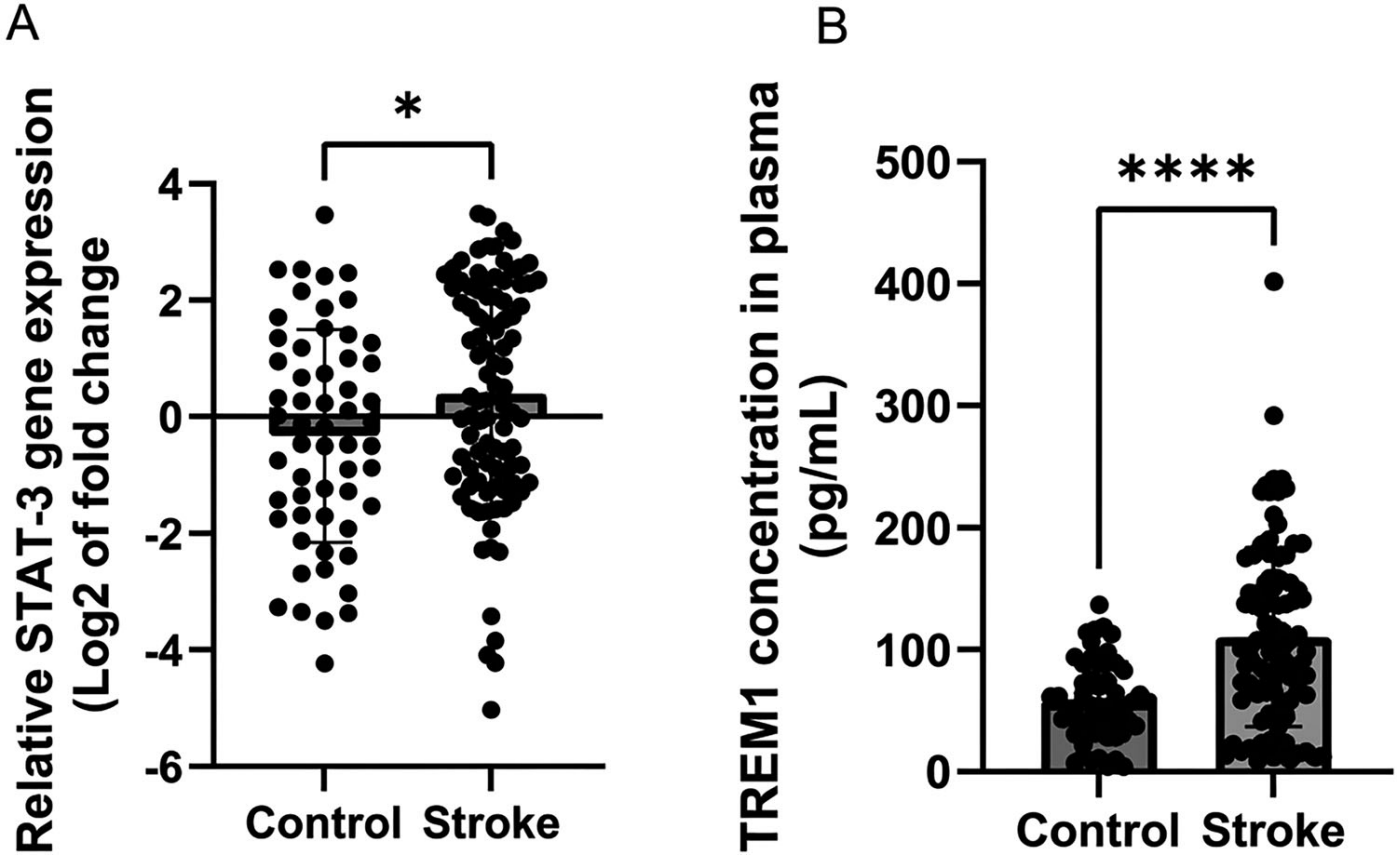
TREM‐1 levels and STAT3 expression in stroke patients and controls. (A) Relative STAT3 mRNA expression, normalized to an internal control and presented as Log_2_ fold change (*p* < 0.0001). (B) The TREM‐1 concentration in plasma in stroke and control (*p* = 0.0238).*p < 0.05, ****p < 0.0001

As shown in Figure 1A,B, plasma concentrations of TREM‐1 and relative gene expression of STAT3 in PBMCs were significantly higher in stroke patients compared to the control group. Specifically, STAT3 mRNA expression was increased by 1.59‐fold in the stroke group relative to controls (*p* < 0.0001). Similarly, the plasma concentration of TREM‐1 was significantly elevated in AIS patients (mean ± SEM, 110.3±4.31 pg/mL) relative to the control group (57.7 ±7.51 pg/mL, *p* = 0.0238).

### 3.3 Sensitivity Analysis

To evaluate whether the inclusion of patients with Ménière's disease in the control group influenced the primary findings, a sensitivity analysis was conducted after excluding these nine participants. Following their removal, plasma TREM‐1 levels remained significantly elevated in the AIS group (mean ± SEM: 110.32 ± 7.52 pg/mL) compared to the remaining controls (57.10 ± 4.34 pg/mL; Welch's *t*‐test: *t* = 6.131, df = 137.96, *p* < 0.001). Similarly, STAT3 mRNA expression remained significantly higher in the stroke group (median [IQR]: 0.3227 [−1.0739 to 2.1986]) relative to the reduced control cohort (median [IQR]: −0.4677 [−1.6392 to 0.9914]; Mann–Whitney *U* test, *p* = 0.027). These findings suggest that the primary findings were not solely driven by the inclusion of these controls.

### 3.4 Subgroup Analysis by Time From Symptom Onset

To evaluate whether the timing of blood sampling within the 7‐day enrollment window influenced biomarker levels, AIS patients were stratified into those sampled within 24 h of symptom onset (<24 h group, *n* = 19) and those sampled after 24 h (≥24 h group, *n* = 77). The median time from symptom onset to blood sampling was 9 h (IQR 4.5–19.5 h) in the <24 h group and 72 h (IQR 34–96 h) in the ≥24 h group.

**FIGURE 2:**
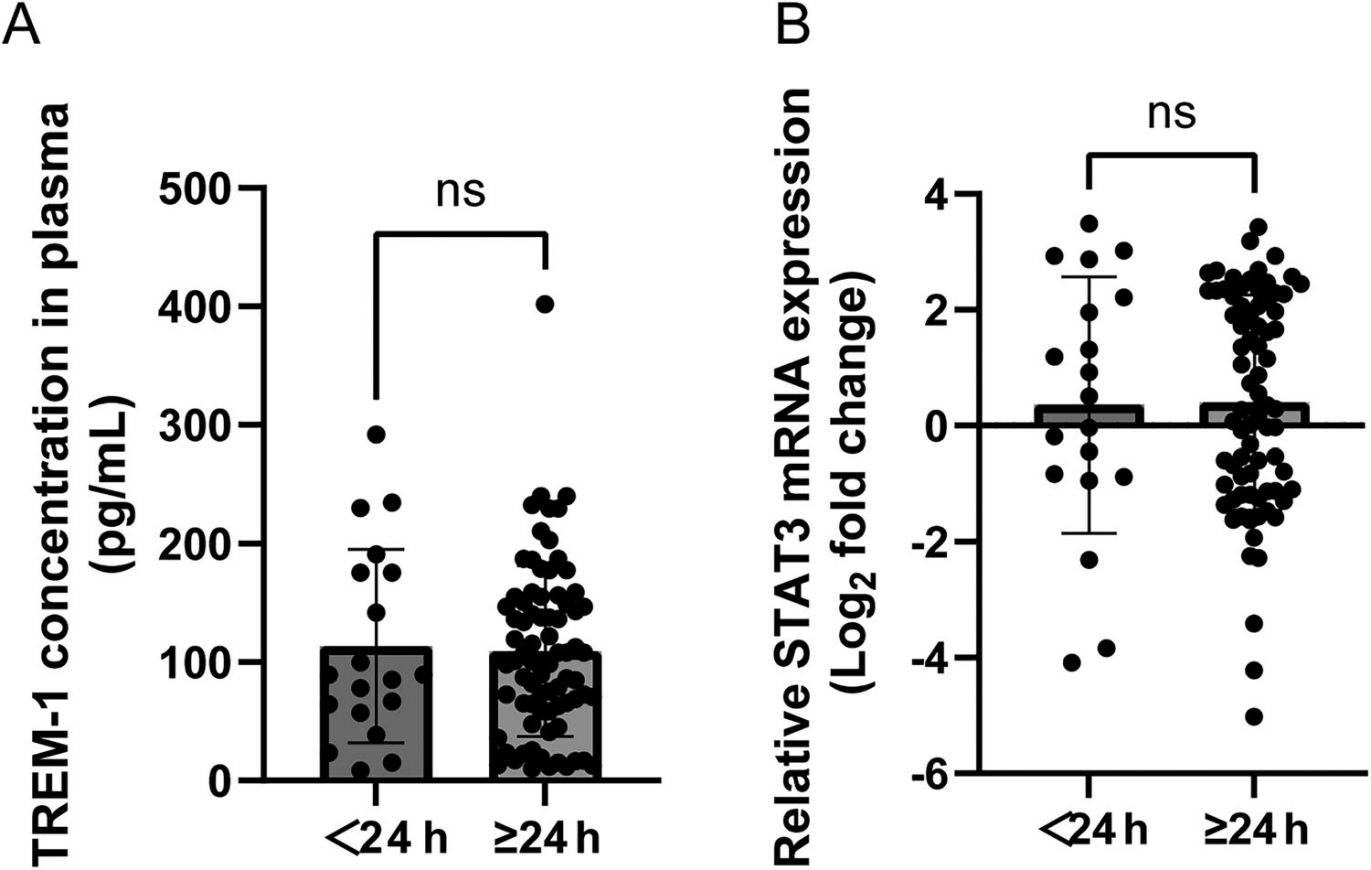
TREM‐1 levels and STAT3 expression according to time from symptom onset. (A) The TREM‐1 concentration in plasma in two subgroups (*p* = 0.8321). (B) Relative STAT3 mRNA expression, normalized to an internal control and presented as Log_2_ fold change (Mann–Whitney *U* test, *p* = 0.850).

As shown in Figure 2A,B, plasma TREM‐1 levels did not differ significantly between the <24 h and ≥24 h groups (mean ± SEM: 113.6 ± 18.76 vs. 109.5 ± 8.21 pg/mL; *t* = 0.2126, df = 94, *p* = 0.8321). Similarly, STAT3 mRNA expression was not significantly different between the two groups (median [IQR]: 0.500 [−0.880 to 2.210] vs. 0.290 [−1.125 to 2.200]; Mann–Whitney *U* = 942.5, *p* = 0.850). These findings suggest that TREM‐1 and STAT3 levels did not differ significantly according to whether blood sampling was performed within or after 24 h from symptom onset.

### 3.5 Correlation Between Biomarkers and Neurological Outcome

**FIGURE 3:**
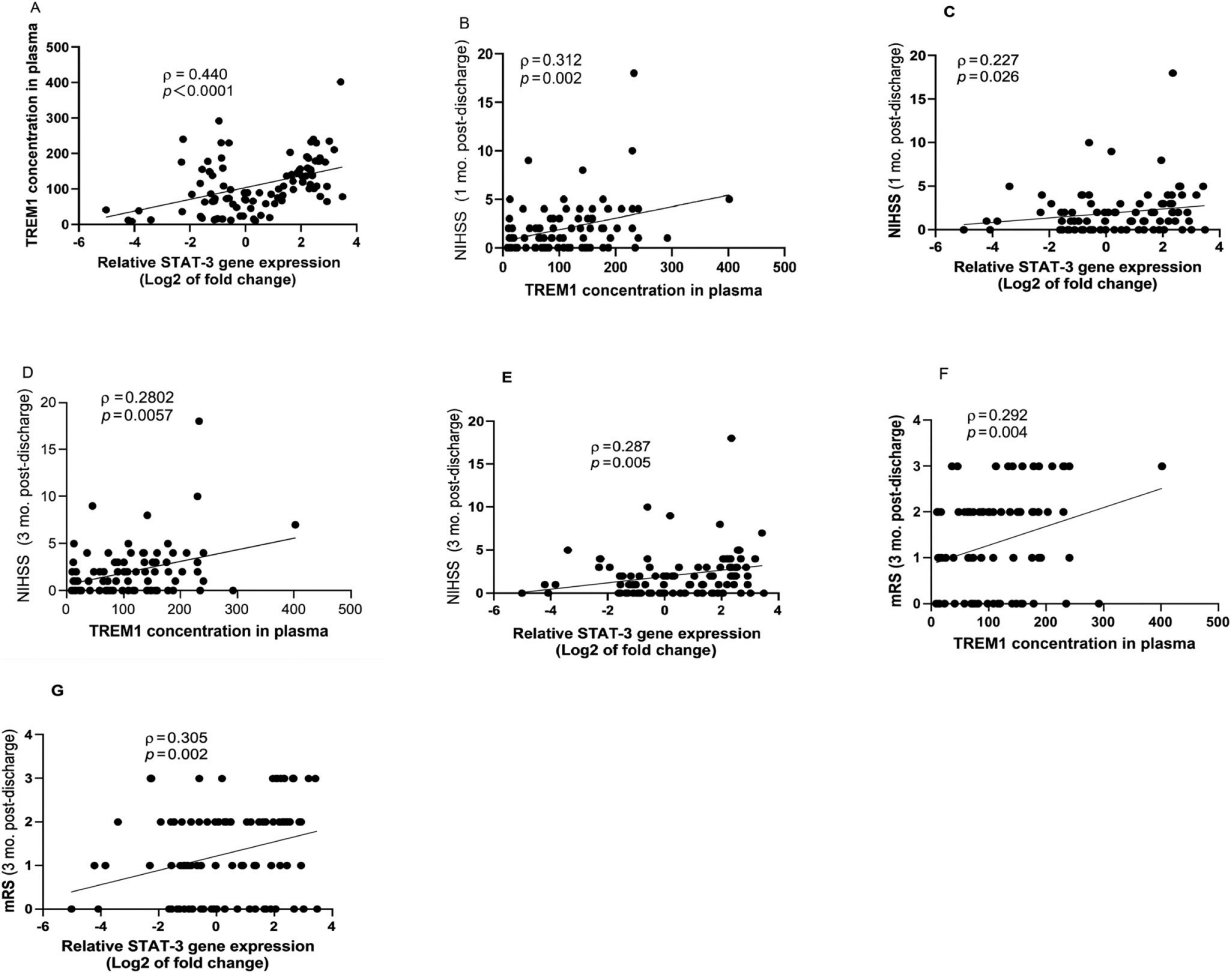
Correlation between TREM‐1 levels/STAT3 expression and outcome. Panels show Spearman correlation data for (A) TREM‐1 levels versus STAT3 expression; (B) TREM‐1 relative to 1‐month post‐discharge NIHSS; (C) STAT3 relative to 1‐month post‐discharge NIHSS; (D) TREM‐1 relative to 3‐month post‐discharge NIHSS; (E) STAT3 relative to 3‐month post‐discharge NIHSS; (F) TREM‐1 relative to 3‐month post‐discharge mRS; (G) STAT3 relative to 3‐month post‐discharge mRS.

A significant positive correlation was observed between plasma TREM‐1 levels and STAT3 expression in PBMCs from patients with AIS (ρ = 0.440, *p* < 0.0001; Figure 3A), supporting the presence of a coordinated inflammatory response. The correlations of both biomarkers with neurological impairment (assessed by NIHSS score) and functional outcome (assessed by the mRS) at multiple time points are summarized in Table 2. Specifically, TREM‐1 levels exhibited stronger correlations with NIHSS scores at both the 1‐month (ρ = 0.312, *p* = 0.002; Figure 3B) and 3‐month (ρ = 0.2802, *p* = 0.0057; Figure 3D) time points compared to STAT3 expression, which demonstrated moderate but statistically significant correlations at 1‐month (ρ = 0.227, *p* = 0.026; Figure 3C) and 3‐month (ρ = 0.287, *p* = 0.005; Figure 3E) assessments. Furthermore, at the 3‐month follow‐up, both TREM‐1 levels (ρ = 0.292, *p* = 0.004; Figure 3F) and STAT3 expression (ρ = 0.305, *p* = 0.002; Figure 3G) were significantly correlated with functional outcome as measured by the mRS, indicating their potential utility in predicting long‐term disability.

**TABLE 2:** Correlations of STAT3 expression and TREM‐1 levels with functional outcomes.

Biomarker	NIHSS at admission	NIHSS at discharge	NIHSS (1 mo.)	NIHSS (3 mo.)	mRS (3 mo.)
STAT3	0.096 (0.350)	0.111 (0.280)	0.227* (0.026)	0.287** (0.005)	0.305** (0.002)
TREM‐1	0.173 (0.093)	0.160 (0.119)	0.312** (0.002)	0.280** (0.006)	0.292* (0.004)

*Note*: Values represent Spearman's rank correlation coefficient (ρ), with corresponding *p* values in parentheses. The abbreviation mo. indicates months post‐discharge.

Abbreviations: mRS, modified Rankin Scale; NIHSS, National Institutes of Health Stroke Scale; STAT3, signal transducer and activator of transcription 3; TREM‐1, triggering receptor expressed on myeloid cells 1.

^*^ *p* < 0.05; **p < 0.01.

### 3.6 Integration of Biomarkers via Principal Component Analysis

**FIGURE 4:**
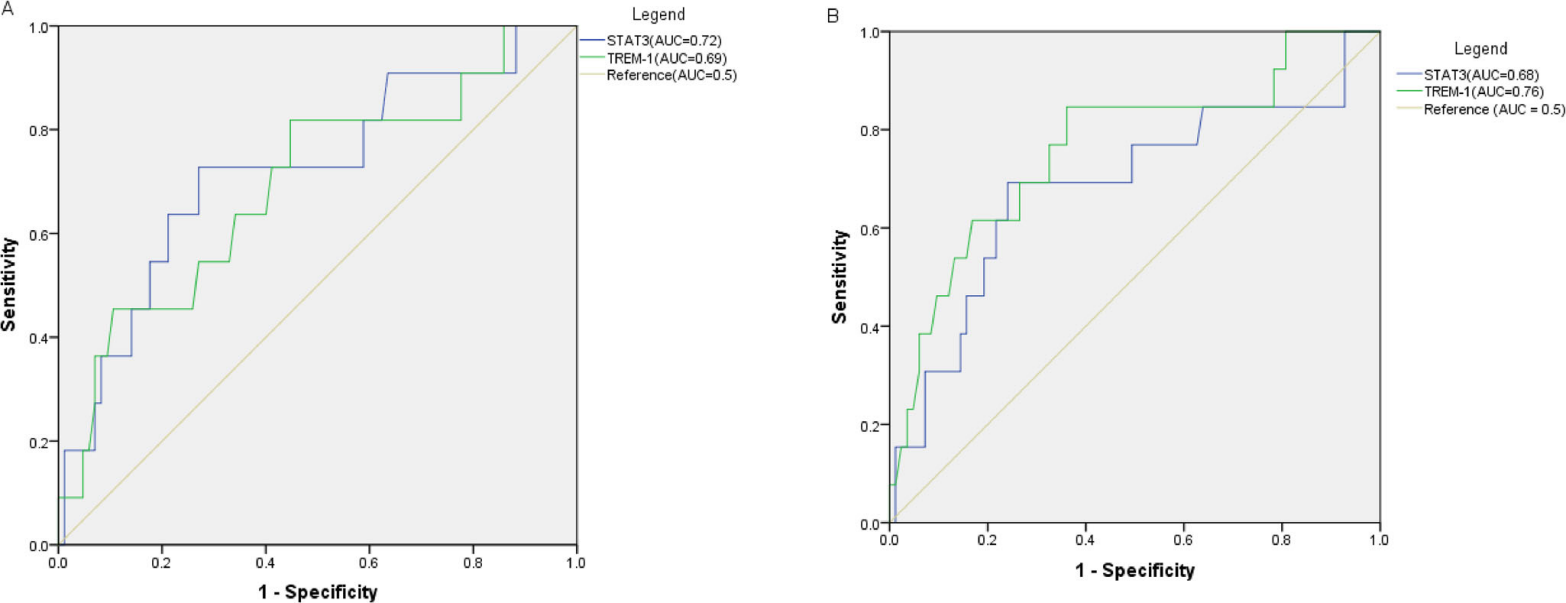
ROC curves for predicting favorable neurological/functional outcome at 3 months. (A) ROC curves of TREM‐1 levels/STAT3 expression for predicting 3‐month neurological outcome. (B) ROC curves of TREM‐1 levels/STAT3 expression for predicting 3‐month functional outcome. The AUC value for each biomarker is displayed on the graph. The diagonal gray line represents the reference line for a test with no discriminative ability (AUC = 0.5). STAT3, signal transducer and activator of transcription 3; TREM‐1, triggering receptor expressed on myeloid cells 1.

To evaluate the prognostic value of the biomarkers, patients were further stratified into two groups based on their NIHSS score (NIHSS ≤ 4 and NIHSS > 4) and mRS score (mRS ≤ 2 and mRS > 2). At both 1 and 3 months post‐discharge, both STAT3 expression and TREM‐1 levels showed significant predictive value for favorable neurological outcomes (NIHSS ≤ 4 or mRS ≤ 2), as assessed by the AUC. Specifically, TREM‐1 levels demonstrated a predictive capacity for an NIHSS score ≤4 (AUC = 0.694) and an mRS score ≤2 (AUC = 0.762). The STAT3 expression demonstrated a predictive capacity for an NIHSS score ≤4 (AUC = 0.72) and an mRS score ≤2 (AUC = 0.684) (Figure 4, Table 3).

**TABLE 3:** ROC analysis for predicting 3‐month stroke outcomes.

Biomarker	NIHSS ≤4 (3 mo.)	mRS ≤2 (3 mo.)
STAT3	0.72 (0.549–0.892)	0.684 (0.507–0.862)
TREM‐1	0.694 (0.518–0.869)	0.762 (0.611–0.913)

*Note*: Data are presented as area under the ROC curve with the 95% confidence interval in parentheses. The abbreviation mo. indicates months post‐discharge.

Abbreviations: mRS, modified Rankin scale; NIHSS, National Institutes of Health Stroke Scale; STAT3, signal transducer and activator of transcription 3; TREM‐1, triggering receptor expressed on myeloid cells 1.

To address multicollinearity between STAT3 expression and TREM‐1 levels and to construct a composite measure, PCA was conducted. A single principal component with an eigenvalue greater than 1, explaining 71.6% of the total variance, was retained. Both STAT3 expression and TREM‐1 levels exhibited strong and approximately equal loadings on this component (loading = 0.846). The resulting component scores were designated as inflammation‐related principal component score (IRPCS) and were used in subsequent logistic regression analyses. In univariate binary logistic regression, STAT3 expression (*χ* ^2^ = 6.150, *p* = 0.013; OR = 1.647, 95% CI [1.04–2.60]; Nagelkerke *R* ^2^ = 0.122), TREM‐1 levels (*χ* ^2^ = 6.382, *p* = 0.012; OR = 1.010, 95% CI [1.002–1.018]; Nagelkerke *R* ^2^ = 0.126), and the composite IRPCS (*χ* ^2^ = 8.949, *p* = 0.003; OR = 2.842, 95% CI [1.32–6.12]; Nagelkerke *R* ^2^ = 0.175) were all significantly associated with poor 3‐month neurological outcome (Table 4).

**TABLE 4:** Comparison of different logistic regression models for prediction performance.

Predictor	Model Sig. (*p*)	Odds ratio (OR)	95% CI for OR	Nagelkerke *R* ^2^	Overall accuracy (%)	Sensitivity (%)
STAT3	0.013	1.647	1.04, 2.60	0.122	88.5	0.0
TREM‐1	0.012	1.01	1.00, 1.02	0.126	89.6	9.1
IRPCS	0.003	2.842	1.32, 6.12	0.175	89.6	9.1

Abbreviations: IRPCS, inflammation‐related principal component score; STAT3, signal transducer and activator of transcription 3; TREM‐1, triggering receptor expressed on myeloid cells 1.

After adjusting for age, sex, smoking, alcohol use, hypertension, diabetes, and onset‐to‐sampling time in a multivariable model, IRPCS remained independently associated with poor outcome (adjusted OR = 2.86, 95% CI [1.27–6.48], *p* = 0.012; Table 5). The overall model fit was modest (Nagelkerke *R* ^2^ = 0.226, Omnibus *χ* ^2^ = 11.717, *p* = 0.164). None of the clinical covariates reached statistical significance in the adjusted model. All predictive models showed low sensitivity (0.0%–9.1%) in identifying patients with poor outcomes, despite high overall accuracy (88.5%–89.6%), reflecting the class imbalance inherent in the study population. In addition, no significant correlation was observed between onset‐to‐sampling time as a continuous variable and plasma TREM‐1 (Spearman ρ = 0.008, *p* = 0.936) or STAT3 expression (ρ = 0.085, *p* = 0.408). This finding, together with the subgroup comparison above, suggests that sampling time within the 7‑day window is unlikely to be a major confounder of the biomarker distributions in this cohort.

**TABLE 5:** Multivariable logistic regression for poor 3‐month neurological outcome (NIHSS >4).

Predictor	Adjusted OR	95% CI	*p* value
IRPCS	2.86	1.27, 6.48	0.012
Age (years)	1	0.91, 1.10	0.96
Sex (male)	0.97	0.16, 5.74	0.972
Smoking	0.54	0.07, 4.44	0.563
Alcohol use	0.63	0.08, 4.85	0.654
Diabetes	1.58	0.31, 8.15	0.584
Hypertension	0.58	0.14, 2.45	0.46
Onset‐to‐sampling time (h)	1	0.99, 1.02	0.983

Abbreviations: IRPCS, inflammation‐related principal component score, Nagelkerke *R* ^2^ = 0.226; Omnibus *χ* ^2^ = 11.717, df = 8, *p* = 0.164; STAT3, signal transducer and activator of transcription 3; TREM‐1, triggering receptor expressed on myeloid cells 1.

## 4 Discussion

This study demonstrates the concurrent upregulation of plasma TREM‐1 and PBMC STAT3 expression in AIS patients. As an amplifier of myeloid inflammatory responses, TREM‐1 enhances signaling through pathways such as Toll‐like receptors (Q. Li et al. 2023), while STAT3 functions as a common downstream node integrating multiple inflammatory signals to regulate cell proliferation, survival, and differentiation (Fan et al. 2013). The significant positive correlation observed between TREM‐1 and STAT3 provides clinical evidence for the TREM‐1–STAT3 inflammatory axis in human disease, extending previous findings predominantly derived from animal models (QIAGEN n.d.). Although neither biomarker was associated with acute stroke severity, their correlation with long‐term functional recovery suggests a role in modulating the chronic inflammatory state that influences neuroplasticity and repair, consistent with evidence that persistent inflammation impedes post‐stroke recovery (Endres et al. 2022). The TREM‐1–STAT3 axis may therefore represent a mechanistic link between the initial immune insult and the trajectory of long‐term recovery.

The composite index IRPCS, derived from TREM‐1 and STAT3 via PCA, outperformed individual biomarkers in its association with poor 3‐month neurological outcome in univariate analysis (OR = 2.84, 95% CI: 1.32–6.12, *p* = 0.008; Nagelkerke *R* ^2^ = 0.175; Table 4). After adjusting for age, sex, smoking, alcohol use, hypertension, diabetes, and onset‐to‐sampling time, IRPCS remained significantly associated with poor outcome (adjusted OR = 2.86, 95% CI 1.27–6.48, *p* = 0.012; Table 5). Onset‐to‐sampling time was not a significant predictor in the adjusted model (OR = 1.00, 95% CI 0.99–1.02, *p* = 0.983), consistent with the subgroup and correlation analyses. However, all models showed very low sensitivity (0.0%–9.1%), indicating that these biomarkers, either individually or combined, cannot reliably discriminate which patients will experience unfavorable recovery. These findings support an association between the TREM‐1–STAT3 axis and post‐stroke outcomes but do not support the use of these biomarkers as stand‐alone clinical prediction tools at present. The low sensitivity reflects the multifactorial nature of stroke recovery, which involves pathways beyond inflammation. Future studies incorporating additional clinical, imaging, and laboratory variables into multivariable prediction models may help improve risk stratification.

Several limitations should be acknowledged. First, the modest sample size (*n* = 96 AIS, *n* = 57 controls) limited the number of covariates that could be reliably included in multivariable models and precluded comprehensive adjustment for confounders such as infarct volume and thrombolysis or thrombectomy status. The unbalanced case‐control ratio (1.7:1) further constrains precision. Second, the control group comprised patients with non‐stroke neurological conditions (BPPV, *n* = 9; PPPD, *n* = 37; TTH, *n* = 4; Ménière's disease, *n* = 9) rather than healthy volunteers. Although individuals with clinically evident systemic inflammatory conditions were excluded, residual confounding related to the underlying neurological disorders cannot be completely ruled out, and future studies incorporating healthy controls or independent validation cohorts are needed. A sensitivity analysis excluding controls with Ménière's disease did not alter the primary findings, supporting the consistency of the observed biomarker elevation in AIS. Third, biomarkers were measured at a single time point after stroke onset. Inflammatory biomarkers may change dynamically during the acute and subacute phases of ischemic stroke. Although biomarker levels did not differ significantly between patients sampled <24 h versus ≥24 h from onset (Figure 2), and no significant correlation was found between onset‐to‐sampling time and TREM‐1 (ρ = 0.008, *p* = 0.936) or STAT3 (ρ = 0.085, *p* = 0.408), this single‐time‐point analysis cannot determine the temporal trajectories or stability of these biomarkers. Serial measurements in future studies are required to define the optimal sampling window and characterize the longitudinal dynamics of TREM‐1 and STAT3 during post‐stroke recovery. Given these limitations, our results should be interpreted as exploratory and require independent validation in larger, multi‐center cohorts. Furthermore, although verbal informed consent was obtained and documented in the study record, written informed consent would provide a more verifiable record and should be adopted whenever feasible in future prospective studies.

## 5 Conclusion

This study provides preliminary evidence that the TREM‐1–STAT3 axis is associated with inflammation and long‐term neurological outcomes in acute ischemic stroke. The composite inflammatory index (IRPCS), derived from TREM‐1 and STAT3 levels, was associated with poor 3‐month functional outcome, even after adjusting for key clinical covariates. However, the low sensitivity of the predictive models (0.0%–9.1%) indicates that IRPCS does not have sufficient discriminative ability for individual‐level clinical risk prediction at this stage. These findings should therefore be regarded as exploratory and hypothesis‐generating rather than as establishing a clinically applicable prognostic tool. Future research should focus on external validation in larger, multi‐center cohorts, longitudinal profiling of these biomarkers, incorporation of additional clinical and imaging variables to improve risk stratification, and investigation of whether targeting the TREM‐1–STAT3 pathway may have therapeutic potential in post‐stroke immunomodulation.

## Author Contributions

**Haohai Lin**: conceptualization, methodology, investigation, formal analysis, writing – original draft. **Yan Dong**: investigation, resources. **Zhangping He**: investigation. **Pingping Li**: resources, supervision, funding acquisition, writing – review and editing. **Chao Qin**: resources. All authors have read and agreed to the published version of the manuscript.

## Funding

This work was supported by the Natural Science Foundation of China (grant No. 82301502), Guangxi Natural Science Foundation Youth Science Fund Project (2025GXNSFBA069034), the First Batch of Medical Youth Reserve Talent Training Project supported by the Health Commission of Guangxi Zhuang Autonomous Region, and the Medical Excellence Talent Development Programme, funded by the Creative Research Development Grant from the First Affiliated Hospital of Guangxi Medical University.

## Ethics Statement

This study was approved by the Ethics Committee of The First Affiliated Hospital of Guangxi Medical University (approval No. 2025‐E0821). All procedures performed in studies involving human participants were in accordance with the ethical standards of the institutional and/or national research committee and with the 1964 Helsinki Declaration and its later amendments or comparable ethical standards.

## Consent

Verbal informed consent was obtained from all participants or their legally authorized representatives, as appropriate. Written consent was not used because the study was conducted in an acute clinical setting, and some patients had neurological deficits or practical difficulties that made written signing burdensome. The study was observational and did not alter routine clinical care. The consent process was conducted by trained research staff, and the participant's or representative's agreement was documented in the study record. Participants were informed that participation was voluntary and that refusal would not affect their clinical care.

## Conflicts of Interest

The authors declare that they have no competing interests.

## Data Availability

The data that support the findings of this study are available on request from the corresponding author, Pingping Li. The data are not publicly available due to privacy or ethical restrictions. Researchers who meet the criteria for access to confidential data may contact Pingping Li at lipingping02@gxmu.edu.cn.
